# Polarization-sensitive optical coherence tomography for estimating relative melanin content of autologous induced stem-cell derived retinal pigment epithelium

**DOI:** 10.1038/s41598-020-64601-4

**Published:** 2020-05-06

**Authors:** Mitsuhiro Matsuzaki, Michiko Mandai, Masahiro Yamanari, Kota Totani, Mitsuhiro Nishida, Sunao Sugita, Tadao Maeda, Naoshi Koide, Seiji Takagi, Yasuhiko Hirami, Noriko Miyamoto, Satoshi Sugiyama, Masayo Takahashi, Yasuo Kurimoto

**Affiliations:** 1 Department of Ophthalmology, Kobe City Eye Hospital, Kobe Hyogo, Japan; 2 Department of Ophthalmology, Kobe City Medical Centre General Hospital, Kobe Hyogo, Japan; 3 Laboratory for Retinal Regeneration, Riken Centre for Biosystems Dynamics Research, Kobe Hyogo, Japan; 4 Tomey Corporation, Nagoya, Aichi Japan; 5 Department of Ophthalmology, School of Medicine, Toho University Oomori Hospital, Tokyo, Japan; 6 Vision Care Inc., Kobe Hyogo, Japan

**Keywords:** Retinal diseases, Imaging and sensing, Regenerative medicine

## Abstract

Transplantation of autologous human induced pluripotent stem cell-derived retinal pigment epithelial (hiPSC-RPE) sheets is a promising therapy for age-related macular degeneration (AMD). As melanin content is a representative feature of healthy RPE, we used polarization-sensitive optical coherence tomography (PS-OCT) to estimate the relative melanin content of RPE in diseased and non-diseased area, and in human iPSC-RPE sheets *in vitro* and *in vivo* by evaluating the randomness of polarization (entropy). Two aged Japanese women, one with neovascular AMD that underwent transplantation of an autologous hiPSC-RPE cell sheet and another with binocular dry AMD, were selected for this study. Entropy value was minimal in cells containing no melanin, whereas that of human RPE and hiPSC-RPE sheets was high. *En face* entropy of the cultured hiPSC-RPE sheet was compared with its grey-scale photo and its values were found to be inversely correlated with the extent of absence of pigmentation *in vitro*. E*n face* entropy maps were compared to colour fundus photographs, fundus autofluorescence images, and fluorescein angiography images from patients. Entropy values of intact and defective RPEs and of iPSC-RPE transplant areas were determined *in vivo* using PS-OCT B-scan images. PS-OCT was found to be applicable in the estimation of relative melanin content of cultured and transplanted RPEs in regenerative medicine.

## Introduction

Age-related macular degeneration (AMD) is a major cause of vision loss in the aging population of developed countries^1^. Although anti-vascular endothelial growth factor (anti-VEGF) therapy for AMD is effective in reducing the activity of choroidal neovascular membrane (CNV) and resolving exudation^2^, it does not restore the lost or damaged retinal cells, including retinal pigment epithelial (RPE) or photoreceptor cells. Since RPE is essential for the survival and function of photoreceptor cells^3^, supplementation by healthy RPE may be a promising approach for treating AMD with anatomical or functional loss of RPE.

As reported previously, a sheet of RPE cells, differentiated from autologous human induced pluripotent stem cells (hiPSCs), was transplanted in a patient with neovascular AMD in September 2014^4,5^. We observed no signs of rejection or tumorigenicity, consistent with the safety of pluripotent stem cell-based transplantation therapies^4,5^. Although we observed stability of the pigment sheet for over 4 years with no recurrence of the background disease and stabilised visual function, we detected no improvement in visual function after surgery, partly because the eye had an advanced AMD at the time of CNV removal and hiPSC-RPE transplantation. However, structure of the choroid was better preserved in the grafted area, during the 4 years post transplantation, compared to that in the surrounding RPE-deficient area, thus suggesting some function performed by the hiPSC-RPE sheet^5^. As RPE cells are an important part of blood-retinal barrier and are essential for supporting photoreceptor survival and function, or for choroidal maintenance^3^, clinical observation of RPE atrophy often precedes diseased ocular environment and photoreceptor cell degeneration. In both pathology and regenerative medicine, monitoring the presence or reduction of healthy RPEs *in vivo* would be important—as an early and sensitive marker—for the pathological event or its recovery. Thus, an objective and straightforward parameter for measuring functional aspects of healthy, diseased, and grafted RPEs is desired.

We had previously demonstrated the degree of pigmentation to be highly correlated with characteristic functions of hiPSC-RPE sheets, including secretion of vascular endothelial growth factor (VEGF) and pigment epithelium-derived factor (PEDF), and transepithelial electrical resistance (TER)^6^; however, it is difficult to objectively and quantitatively evaluate the pigmentation of RPEs *in vivo*, using routine imaging tools such as colour fundus pictures or autofluorescence images.

Polarization-sensitive optical coherence tomography (PS-OCT) provides tissue-specific contrast based on optical polarization properties of cells and tissues^7^. Pircher *et al*. was the first to report that RPE is observed as high polarization scrambling layer in human macula^8^. Degree of polarization uniformity (DOPU)^9^ has been used to segment and observe RPEs along with some unique features of retinal diseases^10–12^. Baumann *et al*. had reported a spatial distribution of DOPU at the RPE layer, with a discussion that DOPU might reflect the degree of melanin content in RPE cells^13^. Later, melanin concentration was shown to correlate with DOPU, using melanin suspensions^14^. Consistently, RPE layer had low depolarizing signals in humans and rats with albinism compared to that in the controls^14–16^, suggesting that melanin granules cause high polarization scrambling in both *in-vivo* imaging and *in vitro*. Yamanari *et al*. introduced a mathematical framework to calculate “entropy”, which indicated the randomness of polarization property in an advantageous way, both physically and mathematically^17^. The entropy was also sensitive to melanin in the iris pigment epithelium^17^ and in RPE^18^, but has not been used for clinical studies yet.

In this study, we first used our prototype PS-OCT to measure entropy in the cells with no melanin and in cultured RPE sheets prepared from foetal and hiPSC-derived RPEs to determine the correlation between entropy value and degree of pigmentation obtained from grey-scale images *in vitro*. Thereafter, we measured entropy in RPEs from three eyes with AMD, including one from a patient transplanted with a hiPSC-RPE sheet after 3 years—in our previously reported clinical trial—to determine the possibility of estimating the relative melanin content of RPEs *in vivo*, in the diseased and intact parts of the eyes with AMD, and in the surgically RPE-depleted areas with or without hiPSC-RPE sheet transplantation^4,5^.

## Results

### *In-vitro* evaluation of RPE pigmentation and entropy by PS-OCT

**Figure 1:**
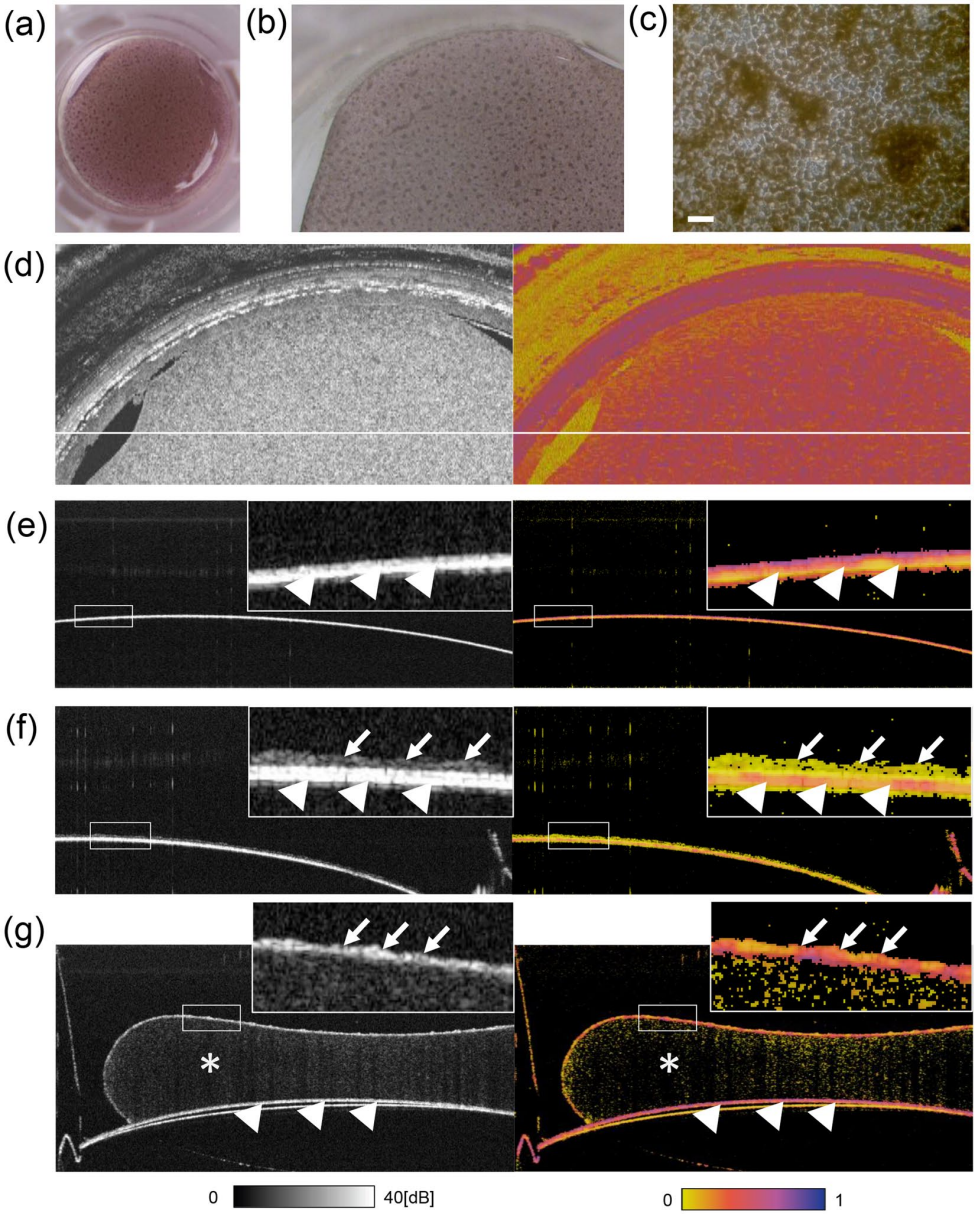
*In-vitro* observation of human foetal RPEs and HEK293T cells devoid of melanin by PS-OCT. Digital photograph (**a**), stereomicroscopic image (**b**), and microscopic image (**c**) of a foetal human retinal pigment epithelial (hRPE) sheet in a transwell presenting a cobblestone-like appearance with pigmented cells. The sheet was used after 71 days of seeding in a transwell. *En face* map of polarization-sensitive optical coherence tomography (PS-OCT) intensity imaging (left) and entropy imaging(right) of the hRPE sheet (**d**). PS-OCT intensity (left) and entropy (right) of cross-sectional B-scan images of the transwell membrane only (**e**), human embryonic kidney (HEK) 293T cells on the membrane as a negative control (no melanin containing cells) (**f**), and the hRPE sheet on collagen gel on the membrane (**g**). In (**d–f**), arrowheads indicate a transwell membrane with high intensity and high entropy; arrows indicate presence of cells; asterisks indicate collagen gel. Scale bar, 50 µm.

A human RPE (hRPE) sheet, prepared from foetal hRPE cells 10 weeks after being seeded on the collagen-coated transwell, is shown in Fig. 1a,b. Figure 1c shows a magnified view of the hRPE sheet with a cobblestone like appearance with a variable degree of pigmentation. Similar *en face* borders of the hRPE sheet were observed in the colour photo (Fig. 1b), OCT intensity image, and entropy image (Fig. 1d).

We first observed hRPE and human embryonic kidney (HEK) 293T cells using PS-OCT, and HEK293T cells with no melanin served as the negative control (Fig. 1e,f). A transwell membrane presented a high-intensity band and exhibited high entropy (Fig. 1e, arrowheads). The cell-like shapes of HEK293T cells were observed on the high-intensity band (insert membrane) by intensity OCT imaging, although showing minimal entropy while the insert membrane showed high entropy (Fig. 1f, cells indicated by arrows and the membrane by arrowheads). The hRPE sheet on collagen gel was observed as a line of cells in an intensity OCT image, exhibiting a mosaic of moderate to high entropy, whereas the insert membrane under the collagen gel consistently exhibited high entropy (Fig. 1g, cells indicated by arrows and the membrane by arrowheads).

**Figure 2:**
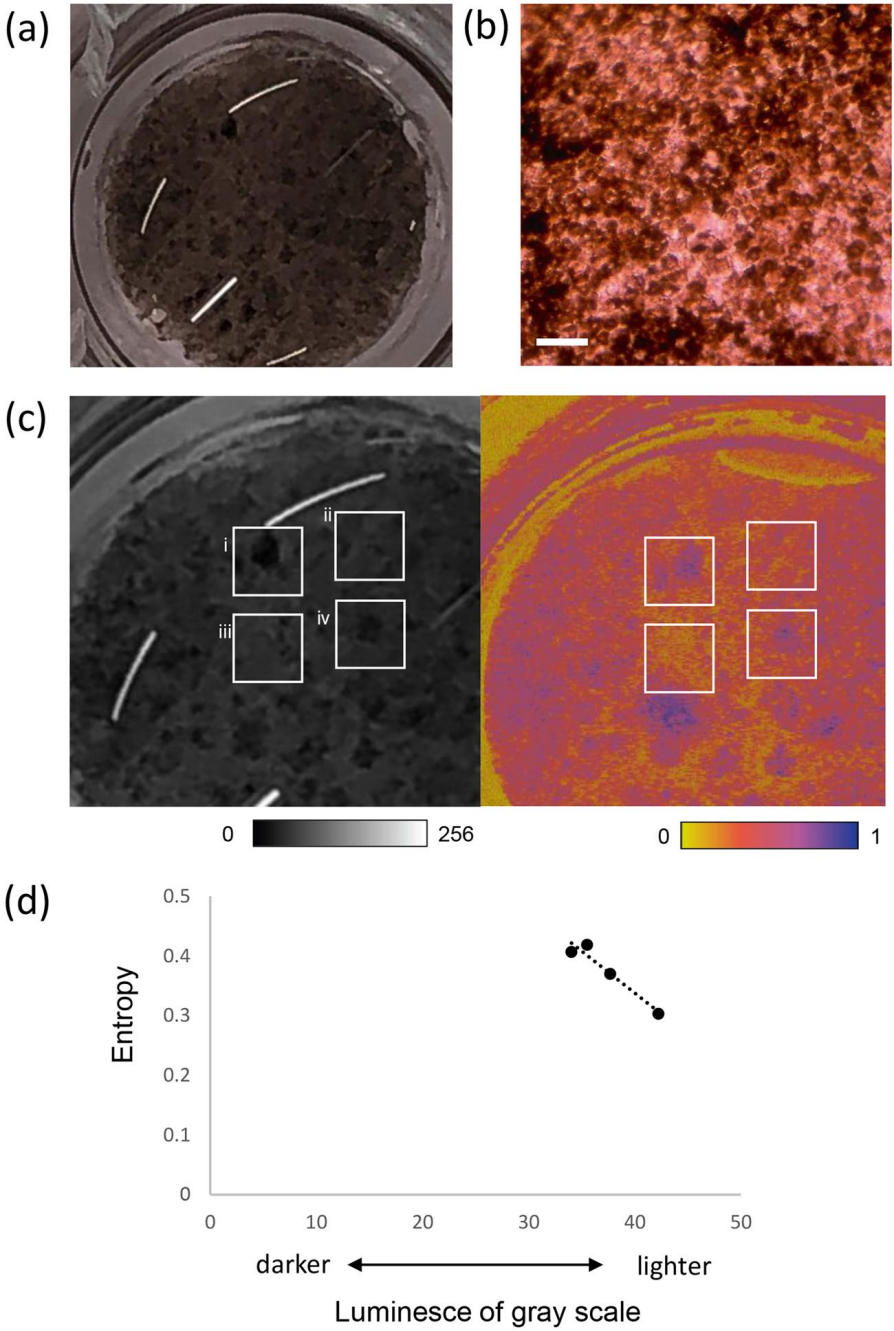
Evaluation of entropy relative to the degree of pigmentation in hiPSC-RPE sheet *in vitro*. Digital photographs (**a**) and micrograph (**b**) of human induced pluripotent stem cell-derived retinal pigment epithelial (hiPSC-RPE) sheets in transwells, showing pigment spots. The sheet was used after 62 days of seeding in a transwell. Enlarged grey-scale image (c, left) corresponding to *en face* entropy map. Polarization-sensitive optical coherence tomography entropy map of a hiPSC-RPE sheet showing variation in entropy values (c, right). Scatter-plot of entropy vs. luminesce of grey-scale (a higher value indicates less pigmentation) in each region of interest (**d**). Scale bar, 50 µm.

We next observed the hiPSC-RPE sheet *in vitro* using PS-OCT. hiPSC-RPEs were consistently differentiated from 253G1HA iPSC line as previously described^4,19^, which showed a typical cobblestone like appearance with tight junctions as labeled by ZO-1 and expressed RPE marker genes in a similar pattern with primary hRPEs (see Supplementary Fig. S1). These hiPSC-RPEs derived from 253G1HA line were also capable of phagocytizing rod outer segments similar to hRPEs (see Supplementary Fig. S2). We had reported earlier that the functional features of hiPSC-RPE sheet, such as secretion of VEGF, PEDF, and TER, increased and the degree of pigmentation was enhanced along with maturation of cells over 2–6 weeks after seeding, and secretion of VEGF/PEDF, seem to further increase after the degree of pigmentation has reached a plateau^19^. The same lot/passage of hiPSC-RPEs that also expressed representative RPE marker genes as provided in supplement (see Supplementary Fig. S3) were seeded on the collagen gel, and a hiPSC-RPE sheet presented patches of dense pigmentation with a cobblestone-like appearance after 9 weeks (Fig. 2a,b). *En face* PS-OCT revealed high-entropy area corresponding to the densely pigmented regions of the hiPSC-RPE sheet (Fig. 2c). We then quantitatively compared the entropy values with the degrees of pigmentation in the hiPSC-RPE sheet. Four square regions of interest (ROIs) from the grey-scale photograph and corresponding regions from the *en face* entropy map were analysed. Grey-scale value for each ROI was used as a parameter for the degree of pigmentation. Mean grey-scale values (higher values indicating less pigmentation) of each area were (i) 35.50 ± 14.64, (ii) 38.82 ± 9.40, (iii) 42.23 ± 9.72, and (iv) 34.04 ± 9.04, respectively. The mean entropy values of each ROI were calculated to be (i) 0.419 ± 0.140, (ii) 0.374 ± 0.097, (iii) 0.303 ± 0.106, and (iv) 0.407 ± 0.132, respectively. Degree of absence of pigmentation (whiteness) was inversely related to the entropy value (Fig. 2d).

### ***In-vivo*evaluation of RPEs in eyes with AMD**

To evaluate if the entropy distribution in the fundus reasonably implies the presence of melanin in RPEs in the patient with AMD, *en face* entropy maps were created by excluding the melanin-rich choroid area (as described in Methods), and compared qualitatively with colour fundus photographs, fundus autofluorescence (FAF), and fluorescein angiography (FA) images from a bilateral AMD case, to observe the relationship between RPE entropy and other imaging modalities.

**Figure 3:**
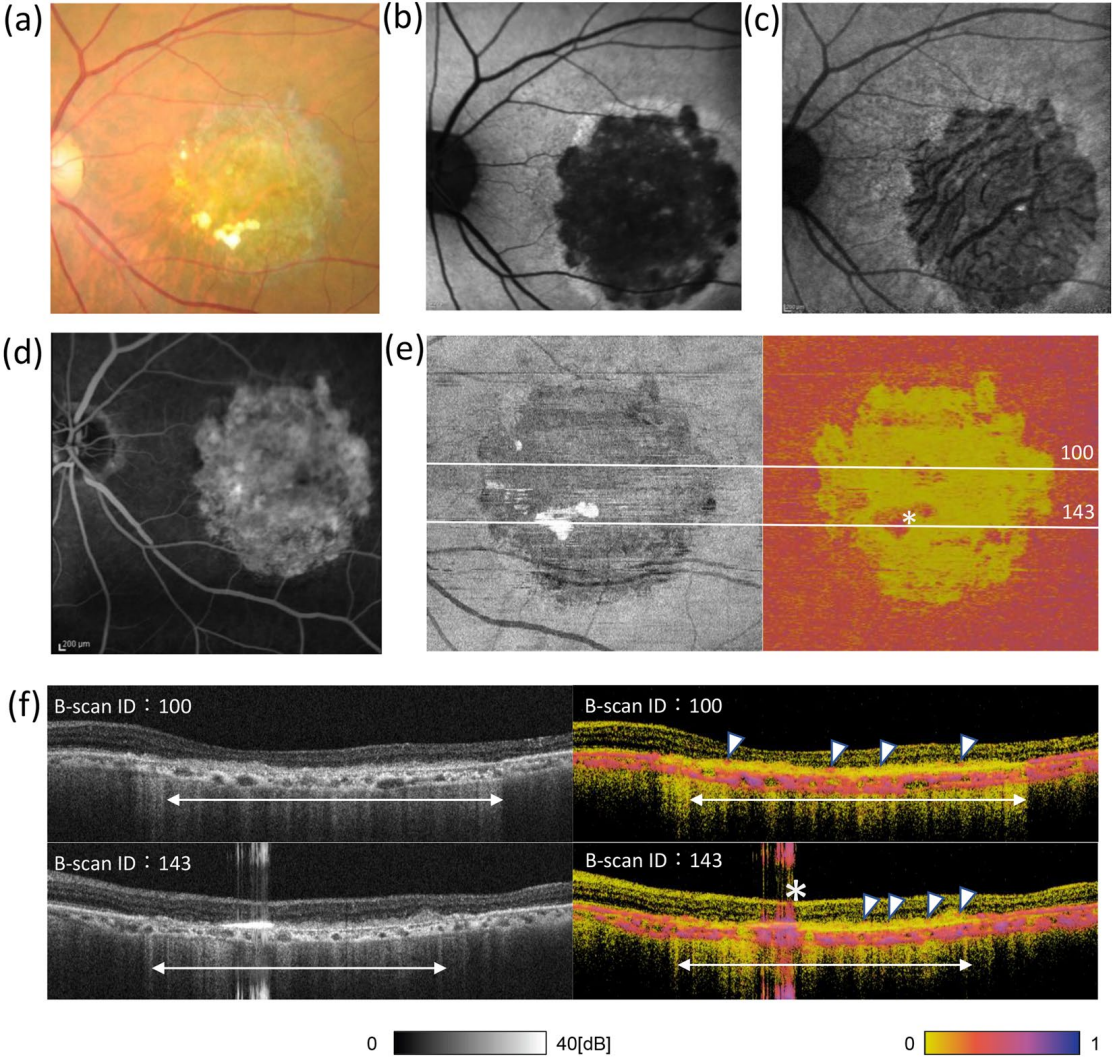
Ophthalmologic and PS-OCT images of the left eye of a patient with AMD. Colour fundus photograph (**a**), fundus autofluorescence (AF) images (**b,c**), and fluorescein angiography image (**d**) of the left eye of a patient with dry age-related macular degeneration. AF images were taken at 532 (**b**) and 787 (**c**) nm. Polarization-sensitive optical coherence tomography intensity (left) and *en face* entropy (right) map excluding the effect of choroidal melanin (**e**), as described in Methods, and cross-sectional B-scan images (**f**) of the left eye of the patient. In (**e,f**), asterisk indicates a signal artefact; arrowheads indicate the possible presence of melanin-containing retinal pigment epithelial cells, showing high entropy on the surface of sub-retinal hyper-reflective mass; double arrows indicate the atrophy area.

In the left eye, a large area of retinal atrophy was observed in the colour fundus photograph (Fig. 3a). It appeared as a hypo-fluorescent area in the FAF images at 532 nm (BAF) and 787 nm (IRAF) (Fig. 3b,c), and as a window defect area in FA images (Fig. 3d). All these images suggested the absence of pigment or healthy RPE in the atrophic area. The *en face* entropy map showed a prominent decrease in entropy corresponding to the atrophic lesion while the lesion is not clearly visible in an intensity map image. (Fig. 3e). The B-scan intensity image (Fig. 3f, left) showed a defect or blurring of the high-intensity RPE band inside the atrophy area (double arrow), whereas PS-OCT detected the possible presence of a few melanin-containing RPEs on the surface of the hyper-reflective sub-retinal mass (Fig. 3f, right, arrowheads). The B-scan image also identified a part of the high-entropy area, presented in the *en face* map, as an artefact (Fig. 3e,f, asterisk).

**Figure 4:**
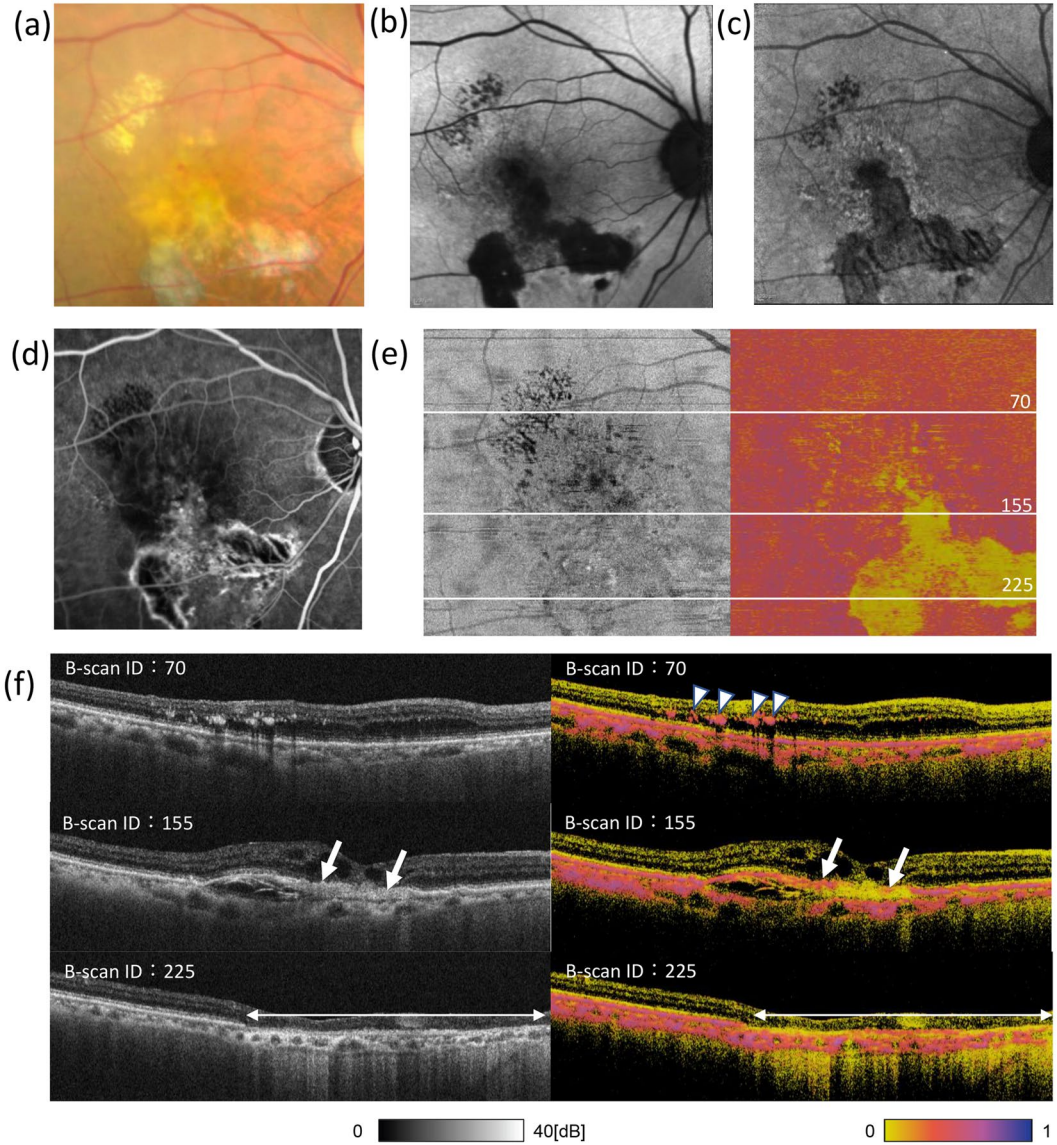
Ophthalmologic and PS-OCT images of the right eye in the same patient as in Fig. 3. Colour fundus photograph (**a**), fundus autofluorescence (AF) images (**b, c**), and fluorescein angiography image (**d**) of the right eye of a patient with dry age-related macular degeneration. AF images were taken at 532 (**b**) and 787 (**c**) nm. Polarization-sensitive optical coherence tomography intensity (left) and *en face* entropy (right) map (**e**), excluding the effect of choroidal melanin, as described in Methods, and cross-sectional B-scan images (**f**) at the lines shown in Fig. 4e. In (**f**), arrowheads indicate intraretinal hard exudate; arrows indicate blurred retinal pigment epithelial lines in the intensity image; double arrow indicates the atrophy area.

In the right eye, an exudative and atrophic region observed in the colour fundus image (Fig. 4a) included a hypofluorescent area, as seen by BAF and IRAF (Fig. 4b,c). The RPE atrophy was observed by FA as either a hyperfluorescent window defect or hypofluorescence when associated with choroidal atrophy (Fig. 4d). The *en face* entropy map exhibited a very low entropy area (Fig. 4e), reasonably similar to the hypofluorescent area by IRAF, which is also known to visualise RPE cells by fluorescence from melanin and/or melanosomes^20^.

Here again, PS-OCT indicated the presence of a fragmented layer of melanin-containing RPEs on the surface of the hyperreflective layer, which appeared as blurred RPE lines in the intensity image (Fig. 4f, arrows). Intraretinal hard exudate also exhibited high entropy, which could be readily annotated by the B-scan entropy image (Fig. 4f, arrowheads).

### PS-OCT analysis of a hiPSC-RPE transplant

**Figure 5:**
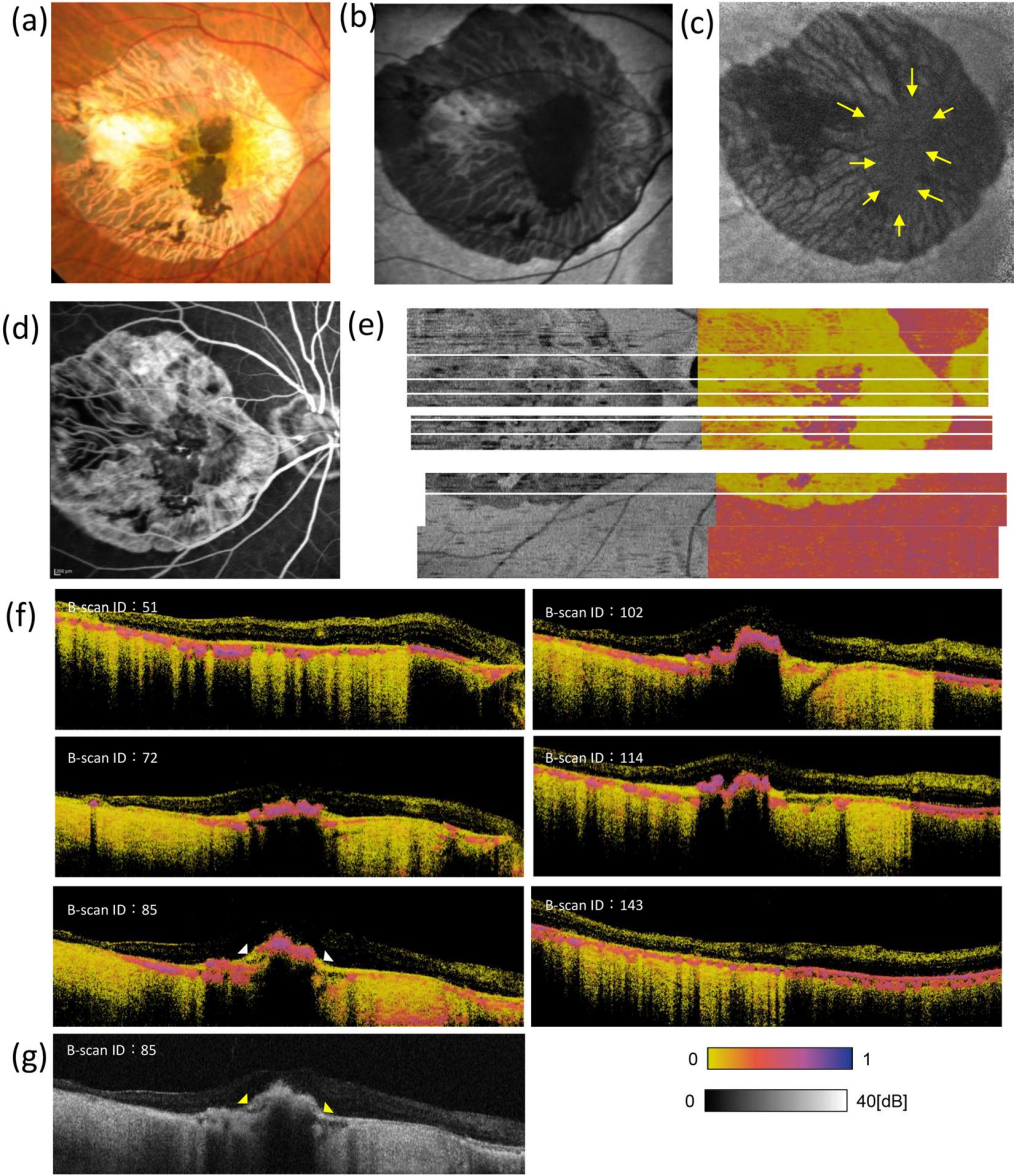
Ophthalmologic and PS-OCT images of the patient that received hiPSC-RPE sheet transplantation. Colour fundus photograph (**a**), fundus autofluorescence (AF) images (**b,c**), and fluorescein angiography image (**d**) of an eye with transplanted autologous human induced pluripotent stem cell-derived retinal pigment epithelial (hiPSC-PRE) cell sheet. AF images were taken at 532 (**b**) and 787 (**c**) nm. Polarization-sensitive optical coherence tomography intensity (right) and *en face* entropy (left) maps of the eye with transplanted hiPSC-PRE (**e**); six lines of cross-sectional B-scan entropy images (**f**); one line of cross-sectional intensity B-scan image (**g**).

Figure 5 shows a colour fundus photograph, a BAF and IRAF image, an early-phase FA image, and an RPE entropy map from the eye that had been transplanted with a hiPSC-RPE sheet 3 years after transplantation. In the transplantation surgery, a large fibrovascular tissue was first removed, and the hiPSC-RPE sheet placed inside the CNV-depleted area, as described previously^4,5^. In the transplanted eye, the hiPSC-RPE sheet inside the large CNV-depleted area was observed in the colour fundus photograph (Fig. 5a). The hiPSC-RPE sheet showed hypo-autofluorescence by BAF (Fig. 5b) while it showed weak autofluorescence by IRAF (Fig. 5c, yellow arrows). The CNV-depleted area also showed weak autofluorescence due to melanin in the choroid. The FA image shows the demarcated window defect area from which the RPE and CNV had been removed as a complex, and hypofluorescence indicated hiPSC-RPE graft sheet and choroidal atrophy (Fig. 5d). The *en face* entropy map (Fig. 5e) shows the hiPSC-RPE sheet as a demarcated high-entropy area. Although the *en face* region of the hiPSC-RPE sheet could be identified by other imaging tools, PS-OCT allowed for quantitative information by entropy values. In the intensity B-scan image, the hiPSC-RPE transplant was hard to distinguish, as contiguous pathological regions (Fig. 5g, yellow arrowheads) showed similar intensities. However, the entropy B-scan image apparently identified the hiPSC-RPE transplant with a high entropy value range, whereas the contiguous pathological region exhibited low entropy (Fig. 5f, bottom left, white arrowheads). Entropy of the hiPSC-RPE transplant was similar to that of peripheral non-diseased RPE (Fig. 5e,f).

### Quantitative evaluation by PS-OCT

**Figure 6:**
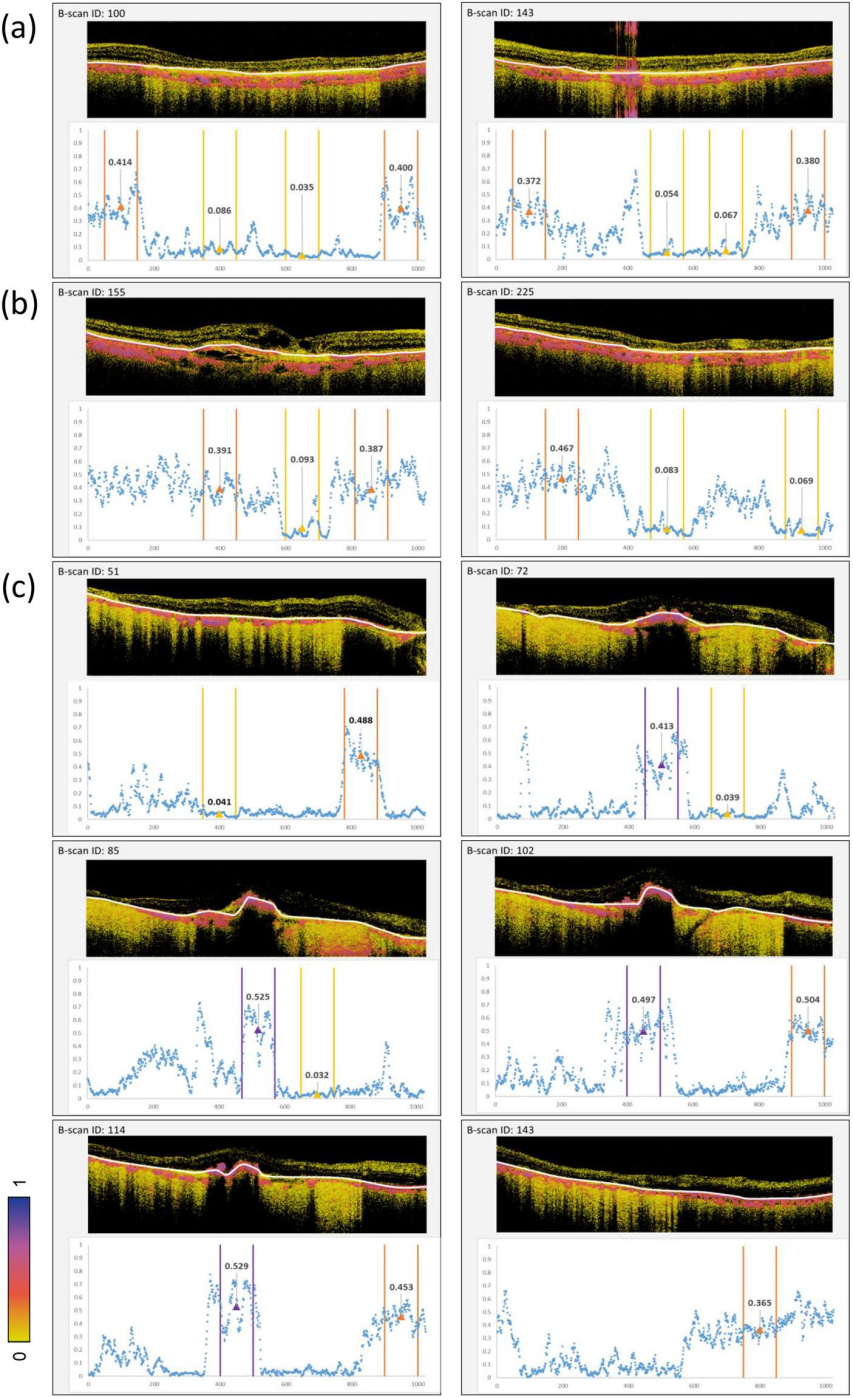
Quantitative evaluation of entropy of RPEs of diseased, healthy, and hiPSC-RPE sheet *in-vivo*. Quantitative evaluation of entropy-related cross-sectional B-scan images from the patient retinas presented in Figs. 3–5. Entropy B-scan images and entropy plot for the left (**a**) and right (**b**) eyes of a patient with dry age-related macular degeneration and for the eye with transplanted autologous human induced pluripotent stem cell-derived retinal pigment epithelial (hiPSC-PRE) cell sheet (**c**). Orange, yellow, and purple ranges in the graph mean the intact RPE, atrophic or defective RPE, and hiPSC-RPE areas, respectively. Mean entropy values are shown as triangles. Measurement area of 100 (width) × 3 (depth) pixels is shown by white lines in B-scan entropy images.

We quantified entropy values of the delineated and labelled ROIs from the entropy B-scan images of the 3 eyes described in Figs. 3–5. The intact RPE, atrophic or defective RPE, and hiPSC-RPE areas were extracted from the delineated entropy graph obtained from each entropy B-scan image. In the eyes of the subject with AMD, the mean entropy values and standard deviation (SD) of the intact RPE area were 0.391 ± 0.092 in the left eye (Figs. 3, 6a) and 0.415 ± 0.086 in the right eye (Figs. 4, 6b). In the RPE atrophic area, the mean entropy values and SD were 0.061 ± 0.034 in the left eye and 0.082 ± 0.058 in the right eye. In the patient with hiPSC-sheet transplantation, the mean entropy values and SD at the hiPSC-RPE transplant area, RPE defective area, and peripheral intact RPE area were 0.491 ± 0.130, 0.037 ± 0.0017, and 0.453 ± 0.085, respectively (Figs. 5, 6c).

## Discussion

Our *in-vitro* PS-OCT observations showed the cultured hRPE- and hiPSC-RPE sheets to have high entropy while no melanin containing cells showed minimal entropy. The degree of pigmentation on grey-scale (a higher value indicating lesser pigmentation) was inversely related to entropy in the hiPSC-RPE sheet. Polarization scrambling is reported to be associated with the concentration of melanin granules in water, using the degree of polarization uniformity^14^. Furthermore, the degree of pigmentation has been reported to be correlated with the maturity of hiPSC-RPE^6^. These results indicate entropy, measured by PS-OCT, to potentially be used for the evaluation of the presence of healthy RPE and maturity and/or functional state of the transplanted hiPSC-PRE. An objective evaluation of the degree of RPE pigmentation is not possible with routine imaging tools, such as colour fundus photography or autofluorescence imaging, since they depend on photographic conditions that are difficult to control, such as background light, performance, and settings of the camera. The present study suggested that entropy values as evaluated by PS-OCT are useful for quantifying the degree of RPE pigmentation *in vivo* more objectively; however, further studies would be required for its validation.

In this study, we first reconstructed *en face* entropy maps by excluding choroidal signals, thereby allowing direct comparison of the high-entropy RPE with other *en face* images. One of the advantages of PS-OCT is its ability to acquire three-dimensional information to analyse B-scan images and volumetrically confirm the origin of the signal, as suggested by Miura *et al*.^21^. Although all of these imaging methods (i.e., colour fundus photography, FAF, and FA) could identify the pigmented RPE in *en face* images, we could further observe the detailed status of RPE cells, such as scattered coverage of possible RPE cells, on the sub-retinal hyper-reflective mass in B-scan images using PS-OCT. Using B-scan images, intra-retinal hard exudates were observed as high entropy materials, which is consistent with previous reports^22^.

Compared to FA images, PS-OCT and FAF images were not affected by choroidal blood flow. Our results showed the *en face* entropy maps to share certain similarities with FAF images. FAF involves imaging based on autofluorescence of the retina. BAF uses 488-nm excitation to detect lipofuscin, mainly bis-retinoid N-retinylidene-N-retinylethanolamine (A2E)^23,24^. IRAF uses 787-nm excitation to detect melanin^20^, similar to the high-entropy pattern of the *en face* entropy map by PS-OCT. Autofluorescence of the graft could not be observed clearly in IRAF of the transplantation subject due to weak background autofluorescence from melanin of the choroid in the area lacking fibrovascular membrane. On the contrary, the *en face* map, after segmentation by PS-OCT, could technically distinguish the melanin of RPE from that of the choroid.

Lastly, entropy values of the transplanted hiPSC-RPE were similar to those of non-lesion peripheral RPE, indicating that the hiPSC-RPE sheet maintains similar melanin content 3 years after engraftment. Although a more precise understanding of the relationship between function and melanin content in RPE *in vivo* remains to be clarified, PS-OCT may be considered an effective tool to objectively monitor the survival and melanin content of transplanted RPEs in regenerative medicine.

## Methods

This prospective observational study was registered and approved by the institutional review board of Kobe City Medical Centre General Hospital and conducted in accordance with the Declaration of Helsinki and its later amendments. The study was registered in the University Hospital Medical Information Network (UMIN) registry before commencement (UMIN000011929, transplantation of iPS cell sheet; UMIN000029060, PS-OCT).

### Preparation of hiPSC-RPE, hRPE, and HEK293T cells

Human iPSC-RPE cells were differentiated from hiPSC line 253G1HA (RIKEN BioResource Centre, Tsukuba, Japan), as previously described^4,19^. Human foetal RPE cells (Lot No. 493461 and 476621, Lonza, Walkersville, MD, USA) and hiPSC-RPE cells were characterised by the expression of RPE marker genes (see Supplementary Fig. S1 and S3) and phagocytosis (see Supplementary Fig. S2). To confirm melanin-specific contrast using PS-OCT *in vitro*, RPE cell sheets were prepared by seeding hRPE and hiPSC-RPE cells at 10^5^ cells/well in 12-well transwells (Corning Costar, Cambridge, MA, USA) coated with collagen gel (Nitta Gelatin, Osaka, Japan), as described previously^4,19^. Cells were cultured in serum-free retinal medium supplemented with basic fibroblast growth factor (10 ng/mL) and SB431542 (0.5 mM, Sigma-Aldrich, St. Louis, MO, USA). The medium was changed every 2 or 3 days. Photomicrographs of RPE sheets were obtained using a stereomicroscope equipped with a digital camera (Olympus Corporation, Tokyo, Japan).

HEK293T cells were obtained from RIKEN BioResource Centre and maintained in high-glucose Dulbecco’s Modified Eagle’s medium (Sigma-Aldrich) supplemented with 10% foetal bovine serum (Biosera, Kansas City, MO, USA), 50 U/mL penicillin (Sigma-Aldrich), and 50 mg/mL streptomycin (Invitrogen, Waltham, MA, USA). HEK293T cells (5 × 10^5^ cells/well) were seeded on collagen-coated insert membrane in a 12-well transwell plate, as described above. The sheet of HEK293T cells was used as a non-pigmented control to evaluate melanin-specific contrast of human RPE cells.

### Patients and clinical examinations

Two patients, whose eyes were investigated, were as follows: a 77-year-old Japanese woman with neovascular AMD, who underwent subretinal transplantation of an autologous hiPSC-RPE cell sheet after removal of the large fibrovascular membrane, as reported previously^4,5^, and an 88-year-old Japanese woman with a large area of RPE atrophy caused by dry AMD in both eyes. Informed consent was obtained from these patients, and procedures were approved by the institutional review board.

During routine clinical examinations, colour fundus photographs were obtained using a TRC 50DX instrument (Topcon Corporation, Tokyo, Japan). FAF and FA images were obtained using a Heidelberg Spectralis HRA + OCT instrument (Heidelberg Engineering, Heidelberg, Germany). For FA imaging, 500 mg/10 mL of sodium fluorescein (Fluorescite, Alcon Pharma, Tokyo, Japan) was injected intravenously for 10 s, followed by a flush of 10 mL sterile 0.9% physiological saline (Otsuka normal saline, Otsuka Pharmaceutical Factory, Tokushima, Japan).

### PS-OCT imaging procedures and quantitative analysis

A clinical prototype of PS-OCT (Tomey Corporation, Nagoya, Aichi, Japan) was developed. A frequency-swept laser (Axsun Technologies, Billerica, MA, USA) with a central wavelength of 1.05 μm, sweep repetition frequency of 100 kHz, and wavelength range of 100 nm was used as the light source. The fibre-based optical interferometer used a depth-encoding unit, for two orthogonally polarized incident lights, and a polarization-diverse detection unit, enabling the measurement of all elements of the Jones matrix. Similar systems had already been demonstrated previously^25–27^. Compared to the measurement of Jones vector or Stokes vector^28^, which has been used successfully for many clinical researches^9,11,12^, measurement of Jones matrix has an advantage in revealing the complete response of the target to any state of polarized light but required a complicated hardware and signal processing. Particularly, Lippok *et al*. showed a significant dependence of the polarization scrambling of melanin on the incident state of polarization^29^, which necessitated the measurement of Jones matrix for unambiguous optical characterisation of melanin. Although DOPU has been used to show the spatial randomness of Jones vector (or Stokes vector derived from it)^9^, there is no direct analogue or extension of DOPU for the Jones matrix. Due to this technical reason, other parameters would be required to characterise the randomness of Jones matrix in general. In our approach, we calculated the noise-bias-corrected entropy of local Jones matrices as a measure of spatial randomness of the polarization property by Cloude-Pottier decomposition^17,18^. Entropy of the Jones matrix, which has been widely used and already established in the field of remote sensing^30,31^, is dimensionless and ranges from 0 (completely uniform) to 1 (completely random polarization). Mathematically, entropy of the Jones matrix is categorised as von Neumann entropy and defined using a rank-4 density matrix that is statistically calculated from the measured Jones matrix. Details of the theory are described in Yamanari* et al.*^17^.

For *in-vitro* experiments, 1024 A-scan × 256 B-scan images were obtained covering 6- × 6-mm areas for cultured hRPE, HEK cells, and hiPSC-RPE sheet, using PS-OCT with attachment. Images of *en face* entropy maps were created from the highest entropy for each A-scan at a depth above the transwell membrane to exclude the transwell membrane from the analysis, because the transwell membrane had intrinsically high entropy due to its porous microstructure.

A colour micrograph of the hiPSC-RPE sheet was converted to an 8-bit grey-scale image using ImageJ (National Institutes of Health, Bethesda, MD, USA), and manually registered to the *en face* entropy map. To investigate the relationship between colour and entropy, four regions of interest (ROIs) with different colour patterns in the hiPSC-RPE sheet were set as 1- × 1-mm squares, in consideration of OCT artefacts and light reflections in the photograph. Subsequently, an average grey-scale value from the photograph of each ROI was evaluated for degree of pigmentation by ImageJ. The mean entropy in each ROI was extracted from the *en face* entropy map. The means and standard deviations of grey scale and entropy values in these ROIs were compared. Assuming that the measured entropy of melanin had a symmetric distribution, mean of the entropy was used as a representative value in the ROI.

Raster-scanned images (1024 A-scans × 256 B-scans) were obtained, covering the macular area of 6- × 6-mm in three eyes from the two patients. Using OCT intensity images, RPE and the graft adjacent to the Bruch’s membrane were delineated. The delineated regions were divided and labelled as intact RPE, atrophic or defective RPE, and hiPSC-RPE areas. Since the graft area appeared to have a certain thickness in the OCT image, we delineated the graft area at its axial centre. This delineation was performed by three ophthalmologists specialised in the retina. An *en face* entropy map was created from the delineated curves of 256 B-scan images for each volume. In addition, entropy was quantitatively evaluated in the intact RPE, atrophic or defective RPE, and hiPSC-RPE areas using representative B-scan images. Mean and standard deviation were calculated based on cross-sectional regions of 100 (width) x 3 (depth) pixels, corresponding to 586 ×12.6 μm, centred at the delineated depths. Four representative regions for each B-scan were analysed as shown in Fig. 6, including hiPSC-RPE transplants and AMD subjects.

## Supplementary information

Supplementary information.
