# Supplementary material for: Polarization-sensitive optical coherence tomography for estimating relative melanin content of autologous induced stem-cell derived retinal pigment epithelium
Source: Sci Rep. 2020 May 6;10:7656. doi: 10.1038/s41598-020-64601-4 (PMC7203130; doi:10.1038/s41598-020-64601-4)
Supplement: Supplementary file 1 — Supplementary information. [file 41598_2020_64601_MOESM1_ESM.pdf]

Supplementary Information

Polarization-sensitive optical coherence tomography for estimating relative melanin content of autologous induced stem-cell derived retinal pigment epithelium

Mitsuhiro Matsuzaki, Michiko Mandai, Masahiro Yamanari, Kota Totani, Mitsuhiro Nishida, Sunao Sugita , Tadao Maeda, Naoshi Koide, Seiji Takagi, Yasuhiko Hirami, Noriko Miyamoto, Satoshi Sugiyama, Masayo Takahashi, Yasuo Kurimoto

A

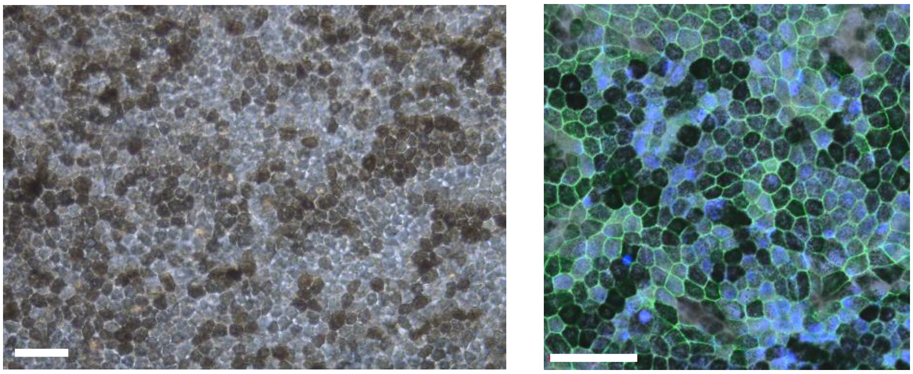

Scale bars 50µm

B

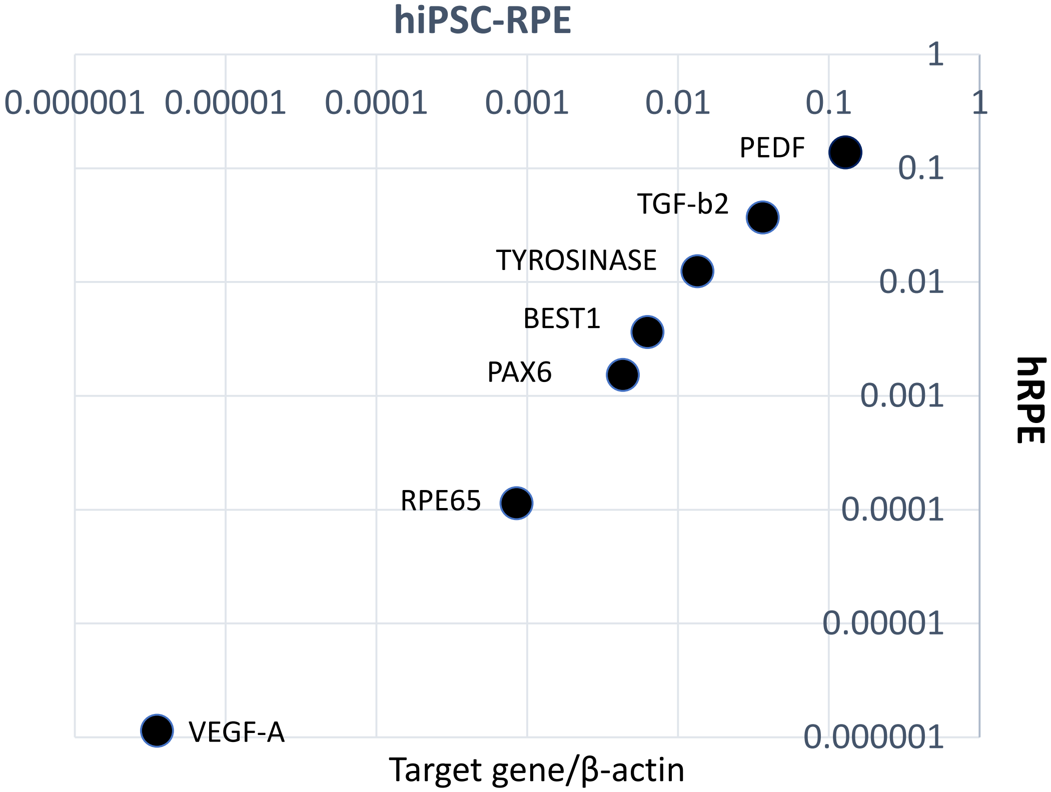

Fig. S1 hiPSC-RPE differentiation and marker gene expression
hiPSC-RPE was differentiated from 253G1HA iPSC line as previously described [1,2]
A A typical cobblestone-like appearance of differentiated RPEs and the presence of tight junction marker ZO-1 by immuno-staining (Invitrogen Anti-ZO-1 Monoclonal (ZO1-1A12), Catalog # 33-9100, 1:500 with Antibody Diluent (Dako# S202230) at 4 ℃ overnight)
B hRPE and hiPSC-RPEs showed a similar expression pattern of RPE marker genes. iPS-RNA was extracted from hRPEs and hRPEs and after cDNA synthesis, target genes were amplified in triplicate by using primers for human PEDF, TGF-b2, RPE65, VEGF-A, tyrosinase, bestrophin-1, Pax6, and β-actin and were analyzed by LightCycler using qPCR Mastermix and Universal ProbeLibrary (all Roche Diagnostics, Tokyo, Japan) as previously described [3] (list of primers and Probes in Table S1). Relative mRNA expression was calculated with Relative Quantification software (Roche Diagnostics) by using an efficiency-corrected algorithm with standard curves and reference gene normalization against that of β-actin [3].

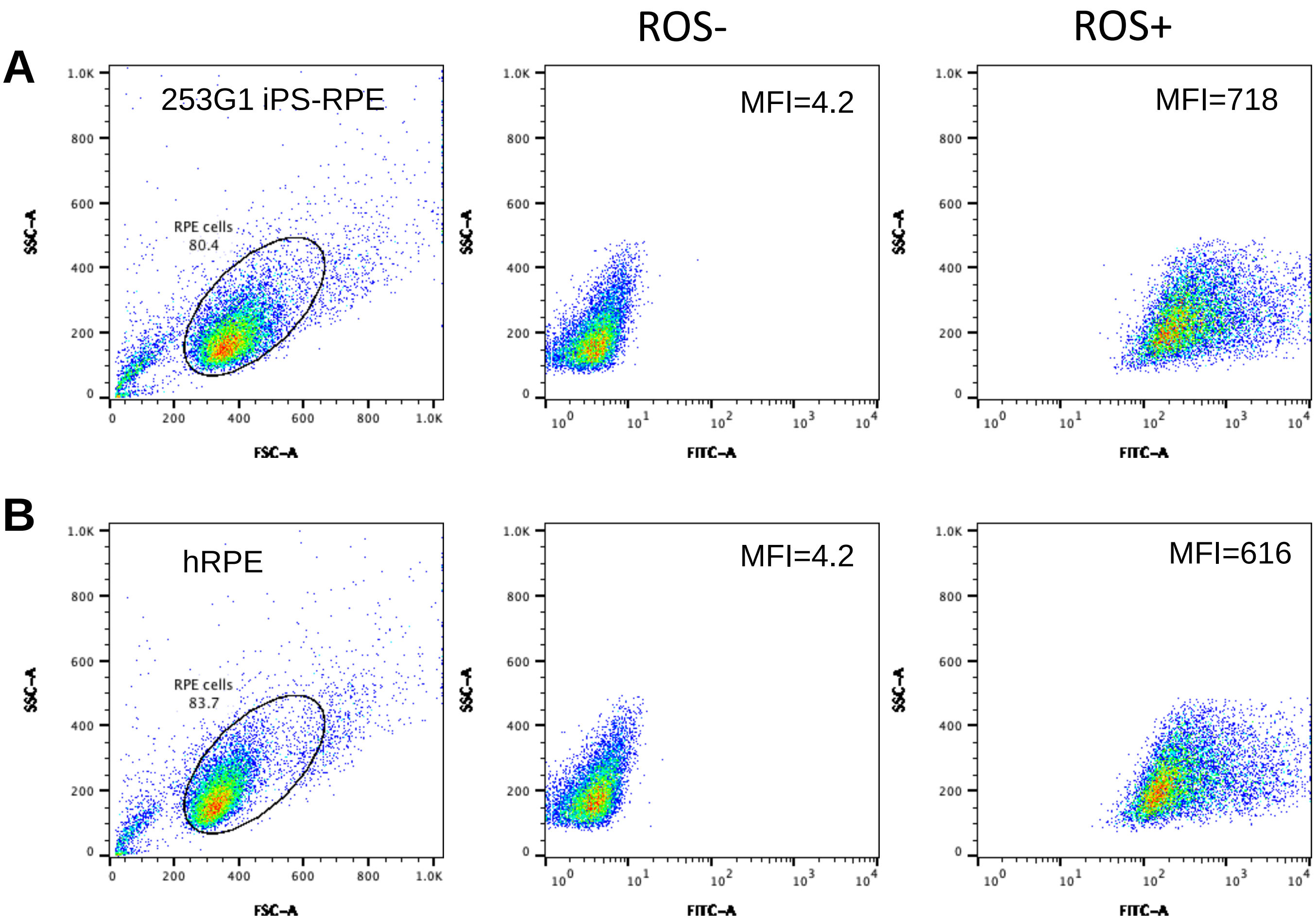

Fig. S2 Phagocytosis of rod outer segments by hRPE cells and hiPSC-RPE cells
Phagocytic function of iPS-RPE (**A**, 253G1HA line) cells and hRPE cells (**B**, Lonza) were examined as previously described[3]. In brief, RPEs were cultured in the presence (right column) or absence (middle column) of FITC-labeled porcine-shed photoreceptor rod outer segments (ROS, 10 μg/cm^2) for 24 hours at 37℃ and FITC positivity was evaluated by flow cytometry analysis. Left panels show the cell gating in FACS. MFI, mean fluorescence intensity.

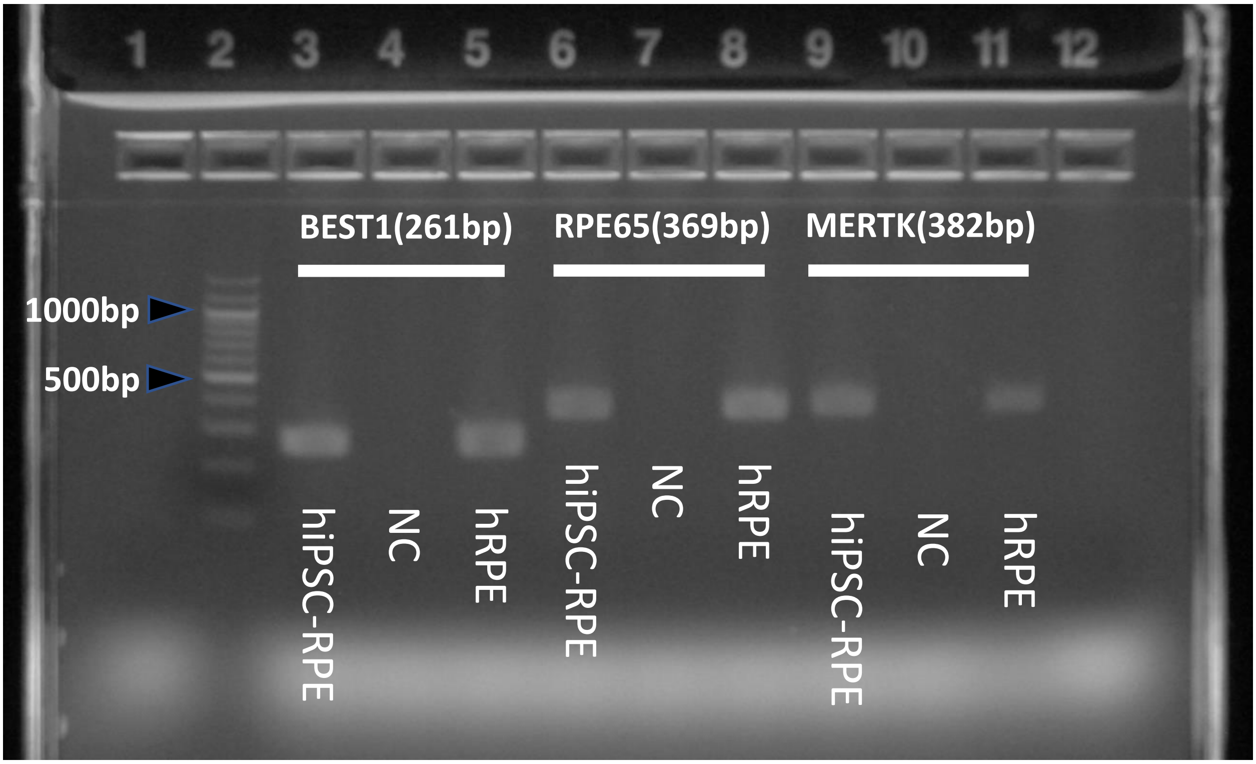

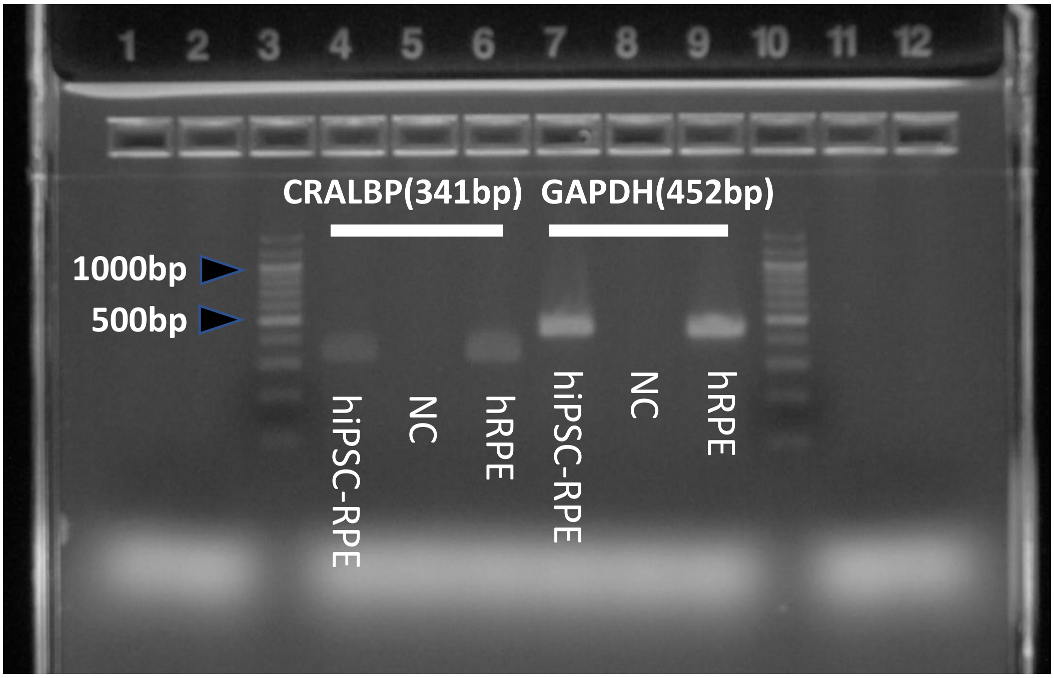

Fig. S3 Marker gene expression by hiPSC-RPE cells from the same lot/passage used in Fig. 2

A part of the cells that were used to make hiPSC-RPE-sheet in Fig. 2 (the same lot) were plated on another dish and stocked upon reaching confluency. RNA was extracted from these hiPSC-RPE cells and was checked for RPE marker gene expressions with hRPE (Lonza) as a control by reverse transcription polymerase chain reaction as previously described(1,2). The pairs of primers used here for each gene are listed on Table S2. NC negative control with water template.

Table S1 List of primers and probes for RPE marker genes used in Fig. S1

	Forward Primers	Reverse Primers	Probe#*
PEDF	gtgtggagctgcagcgtat	tccaatgcagaggagtagca	#57
VEGF-A	cgcaagaaatcccggtataa	aaatgctttctccgctctga	#1
TGF-b 2	ccaaagggtacaatgccaac	cagatgcttctggatttatggtatt	#67
RPE65	caatgggtttctgattgtgga	ccagttctcacgtaaattggcta	#84
Best1	tcttcacgttcctgcagttc	tcctctccaaaggggttgat	#41
Pax6	ggcacacacacattaacacactt	ggtgtgtgagagcaattctcag	#9
Tyrosinase	gctgccaatttcagctttaga	ccgctatcccagtaagtgga	#47
beta-actin	ccaaccgcgagaagatga	ccagaggcgtacagggatag	#64
	probe* probes from the Roche Universal Probe Library		

Table S2 List of primers for RPE marker genes used in Fig. S3

	primer sequence
BEST1-F	TAGAACCATCAGCGCCGTC
BEST1-R	TGAGTGTAGTGTGTATGTTGG
RPE65-F	TCCCCAATACAACTGCCACT
RPE65-R	CCTTGGCATTCAGAATCAGG
CRALBP-F	GAGGGTGCAAGAGAAGGACA
CRALBP-R	TGCAGAAGCCATTGATTTGA
MERTK-F	TCCTTGGCCATCAGAAAAAG
MERTK-R	CATTTGGGTGGCTGAAGTCT
GAPDH-F	ACCACAGTCCATGCCATCAC
GAPDH-R	TCCACCACCCTGTTGCTGTA

References

1 Kamao, H. *et al.* Objective evaluation of the degree of pigmentation in human induced pluripotent stem cell-derived RPE. *Invest. Ophthalmol. Vis. Sci.* **55**, 8309-8318 (2014).

2 Mandai, M. *et al.* Autologous induced stem-cell-derived retinal cells for macular degeneration. *N. Engl. J. Med.* **376**, 1038-1046 (2017).

3 Sugita, S. *et al*. Inhibition of T-cell activation by retinal pigment epithelial cells derived from induced pluripotent stem cells. *Invest Ophthalmol. Vis. Sci.* **56**,1051-1062 (2015).
